# Natural Bio-Sourced Additives for Bread Technology Improvement and Highly Nutritive Products

**DOI:** 10.3390/foods15030413

**Published:** 2026-01-23

**Authors:** Nicoleta Platon, Oana Cristina Pârvulescu, Vasilica Alisa Aruș, Ana Maria Georgescu, Mihaela Silion, Anca Miron, Gabriela Muntianu, Ana Maria Roșu, Petrica Iancu, Abdelkrim Azzouz

**Affiliations:** 1 Department of Chemical and Food Engineering, “Vasile Alecsandri” University of Bacau, 157 Calea Marasesti Street, 600115 Bacau, Romania; nicoleta.platon@ub.ro (N.P.); ana.georgescu@ub.ro (A.M.G.); muntianu.gabriela@ub.ro (G.M.); ana.rosu@ub.ro (A.M.R.); 2 Department of Chemical and Biochemical Engineering, National University of Science and Technology POLITEHNCA Bucharest, 1-7 Polizu Street, 011061 Bucharest, Romania; oana.parvulescu@upb.ro; 3 Department Physics of Polymers and Polymeric Materials, “Petru Poni” Institute of Macromolecular Chemistry, 41A Grigore Ghica Voda Alley, 700487 Iasi, Romania; silion.mihaela@icmpp.ro; 4 Pambac S.A. Bacau, 14 Calea Moinesti Street, 600281 Bacau, Romania; petronela_anca@yahoo.com; 5 Department of Chemistry, University of Quebec in Montreal, Montreal, QC H3C 3P8, Canada; e-azzouz.a@uquam.ca

**Keywords:** bread dough properties, central composite design, hydrolyzed collagen, konjac glucomannan

## Abstract

Hydrolyzed collagen (HC) and konjac glucomannan (KGM) were used as additives in non-frozen and frozen doughs (NFDs and FDs). Both additives were characterized using specific techniques, i.e., SEM-EDX, MALDI-TOF MS, TGA, and DSC analyses. Rheological analysis of NFD samples was performed using a Chopin Mixolab Profiler. According to a central composite design (CCD), two sets of twelve experiments were conducted to evaluate the influence of percentages of HC and KGM in the mixture of flour and both additives (*c_HC_* = 0.79–2.21% and *c_KGM_* = 0.79–2.21%) on the porosity (*PO* = 58.96–78.76%), humidity (*HU* = 42.51–45.60%), electrical conductivity (*EC* = 2.06–2.29 μS/cm), and pH (*pH* = 5.5–5.9) of bread samples prepared from NFD and FD. The freezing led to a significant decrease in *PO* and *pH*, as well as a significant increase in *HU*, whereas its effect on *EC* was not statistically significant. The highest values of response variables that were significantly affected by the process factors, i.e., *PO_FD_* = 70.8%, *pH_FD_* = 5.6, and *pH_NFD_* = 5.9, were obtained in the center point runs (*c_HC_* = *c_KGM_* = 1.50%). For bread samples prepared from FD, the mold development process began approximately four days later than for those prepared from NFD. Bread samples produced from FD and NFD samples in the center point runs showed a low rate of mold formation.

## 1. Introduction

In the bakery industry, a main problem is the growth of mold on bread [1]. The rate of occurrence of this phenomenon depends on the recipe as well as on the operating and storage conditions. Another problem is the presence of ice crystals in frozen bread dough [2,3]. Nowadays, frozen bread doughs are in greater demand in the market because their shelf life is longer compared to that of fresh (non-frozen) bread doughs. Ice crystal formation can significantly influence the structure and properties of frozen dough as well as the final quality of the resulting product. The challenge is to find the most efficient, sustainable, and environmentally friendly ingredients for baked products [1,2,3]. Several studies have investigated the potential of improving the quality of frozen food products by integration of a variety of additives, e.g., hydrocolloids, polyols, cold-active enzymes, and natural deep eutectic solvents (NADESs) [1,2,3].

In recent years, the application of ice-binding proteins (IBPs) has emerged as a promising strategy to decrease the negative effects of freezing on the quality of frozen food products. IBPs can prevent ice recrystallization (the process by which existing ice crystals grow in size), thus helping to protect food products during freezing and frozen storage, ultimately improving their quality and shelf life [4]. Recent studies have highlighted that the use of ice-bonding collagen peptides, e.g., hydrolyzed collagen (HC) from bovine sources, can bring advantages to the bakery industry [5,6].

Due to its special characteristics, including its neutral odor, transparency, low levels of allergenic substances, emulsifiers, and stabilizers, HC is used as an ingredient in various food products [7,8,9]. HC is composed of bioactive peptides and amino acids, which contribute to its health-promoting properties [10]. Collagen of mammalian origins is still predominant on the market due to its low cost and high availability, although certain aspects, including vegan diets and religious restrictions, limit its use [11]. Collagen and its hydrolysates can be safely used in the food industry as natural antioxidants [12]. Studies on bovine collagen have highlighted that its amino acids, e.g., glycine, proline, alanine, histidine, glutamic acid, aspartic acid, and arginine, have anticancer effects [13]. The composition of collagen varies depending on its source, which can influence its functional properties and applications, including in the bakery industry [14]. By using HC, the freezable water content in frozen dough can be reduced by altering the migration and distribution of water molecules [2,4,5,6].

Konjac glucomannan (KGM) is a high-molecular weight water-soluble neutral polysaccharide obtained from tubers of the konjac plant [15]. KGM has D-glucose and D-mannose units at a molar ratio of 1:1.6–1:1.4. It is an indigestible dietary fiber, proven to be effective in modifying intestinal microbial metabolism, reducing weight and cholesterol, and it has been approved by the FDA as a calorie-free food ingredient [16]. Besides these health-promoting benefits, KGM offers great potential for applications in food and pharmaceutical industries for thickening, texturizing, gelling, and water imbibing. The addition of dietary fibers can improve the physicochemical characteristics of the dough and the nutritional value of the final product [17].

Although HC and KGM have been investigated individually as functional ingredients in bakery products, information on their combined effects on dough and bread characteristics has not been reported in the related literature. On the one hand, HC contributes to improving the protein profile and textural properties of bread [7,9,11,18,19]. On the other hand, KGM provides soluble fibers, reduces the glycemic potential of the product, increases water retention capacity, and influences the structure of the gluten network [17,20,21,22]. In the process of dough formation, a three-dimensional gluten network is formed through hydrogen bonds, disulfide bonds, and non-covalent interactions, which wraps starch granules, fats, and other compounds inside [23]. The simultaneous incorporation of HC and KGM into dough could have significant beneficial effects on dough and bread characteristics.

In the present study, HC of a bovine origin and bio KGM were used as low-cost and biocompatible substitutes to the conventional additives in the bread receipt. The influence of the concentrations of HC and KGM additives in the non-frozen and frozen doughs (NFDs and FDs) on the bread physicochemical properties and shelf life was evaluated. Moreover, the additives and doughs were characterized using specific techniques, i.e., scanning electron microscopy (SEM)-energy-dispersive X-ray spectroscopy (EDX) analysis, matrix-assisted laser desorption ionization-time of flight mass spectrometry (MALDI-TOF MS) analysis, thermogravimetric analysis (TGA), differential scanning calorimetry (DSC) analysis, and rheological analysis using a Chopin Mixolab Profiler.

## 2. Materials and Methods

### 2.1. Materials

The materials used to obtain bread samples were wheat flour (WF), fresh yeast (FY), sunflower oil (SO), salt (SA), water, bovine HC, and bio KGM powder additives in different proportions (Table 1). Samples S1–S12 were formulated according to a central composite design (CCD) with two independent variables (factors), i.e., the percentages of HC (0.79–2.21%) and KGM (0.79–2.21%) in the mixture of WF, HC, and KGM. The experimental design included four center point runs. Sample S0, prepared without HC and KGM, was used as a control and was not included in the CCD model.

### 2.2. Physicochemical Characterization of HC and KGM Additives

#### 2.2.1. SEM-EDX Analysis

Surface morphology and elemental composition of HC and KGM additives were evaluated by SEM-EDX analysis using a Verios G4UC scanning electron microscope (Thermo Scientific, Pardubice, Czech Republic) equipped with EDX spectroscopy analyzer (Octane Elect Super SDD detector, AMETEK EDAX, Mahwah, NJ, USA). The samples were coated with 6 nm platinum using a Leica EM ACE200 sputter coater (Leica Microsystems, Wetzlar, Germany). SEM investigations were performed using a backscatter electron detector MD (mirror detector) at accelerating voltage of 15 kV.

#### 2.2.2. MALDI-TOF MS Analysis

MALDI-TOF MS analysis was conducted to molecularly characterize HC and KGM, thus providing essential information about their quality and structure. The experiments were performed on a Bruker RapifleX MALDI-TOF/TOF (Bruker Daltonics, Bremen, Germany) equipped with a Smartbeam 3D laser (Bruker Daltonics, Bremen, Germany). The instrument was operated in positive reflectron mode (*m*/*z* = 400–8000). The spectra were acquired and processed using FlexControl Version 4.0 and FlexAnalysis Version 4.0 software (Bruker, Bremen, Germany). The samples of HC and KGM were dissolved in a solution containing acetonitrile and 0.1% trifluoroacetic acid in deionized water in a 1:1 *v*/*v* ratio, then were spotted on the MALDI target plate, sandwiched with a saturated solution of matrices, i.e., α-cyano-4-hydroxycinnamic acid (CHCA) or sinapinic acid, followed by drying. Additionally, each sample was mixed with each of the matrices in a 1:2 *v*/*v* ratio, separately, and vortexed. These resulting samples were spotted on the MALDI target plate and dried at room temperature before MALDI-TOF MS analysis. The accelerating voltage was set at 20 kV in positive ion mode, and the digitizer at 1.25 GHz. The laser frequency was set at 10 kHz and the laser power at 50% to 70% of the maximum. Mass calibration was performed using a peptide mixture standard solution (Bruker Daltonics, Bremen, Germany).

#### 2.2.3. TGA and DSC Analysis

TGA and DSC analysis were performed to study the thermal behavior and stability of HC and KGM. The thermal stability of materials was evaluated using a Discovery TGA 5500 (TA Instruments, New Castle, DE, USA) by measuring the mass loss as a function of temperature. Samples of 6 mg were placed in platinum crucibles and heated in nitrogen atmosphere in the temperature range 30–700 °C, using a heating rate of 10 °C/min. DSC analysis was performed with a DSC 200 F3 MAIA (Netzsch, Selb, Germany) device. The heating rate was 10 °C/min (from 25 °C to 250 °C), under an atmosphere of dry nitrogen, at a flow rate of 50 mL/min. The data were processed with the NETZSCH PROTENS 4.2 software. *T_onset_* (the temperature at which the beginning of a mass change was observed), *T_peak_* (the temperature at the maximum decomposition rate), *T_end_* (the temperature at which the main decomposition process was completed), and mass losses corresponding to *T_peak_* were relevant parameters used in TGA and DTG to compare the samples.

### 2.3. Production of Dough and Bread Samples

Twelve samples of fresh (non-frozen) fermented dough (NFD) and twelve samples of frozen fermented dough (FD) were prepared by adding HC and KGM, according to the production recipe presented in Table 1. FD samples were obtained as follows: 30 min after dough fermentation, the samples were packed in hermetic bags, placed in a static freezer, and frozen at −18 °C. Thawing of FD samples was performed at room temperature for 21 h. One control NFD sample and one control FD sample were prepared without the addition of HC and KGM. NFD and FD samples were abbreviated S0, S1…S12 in Table 1, where S0 was the control dough sample.

The bread samples were prepared from NFD and FD samples using a TEFAL bread machine (Groupe SEB, Rumilly, France, kneading time: 15 min; fermentation time: 30 min; baking time: 50 min). After baking, the bread samples were tagged and left for 3 h at room temperature for cooling, and then prepared for the different analyses [24].

### 2.4. Characterization of Dough Samples

Rheological behavior of NFD samples under mixing, heating, and cooling conditions was determined using a Chopin Mixolab Profiler (Chopin Technologies, Villeneuve-la-Garenne, France) [25]. The results were presented as Mixolab curves and Profiler graphs. Each Mixolab curve had five characteristic points, i.e., C1–C5, corresponding to different stages of dough processing, i.e., C1 was related to mixing and development of dough, C2 to the mechanical and heating effects on the gluten strength, C3 to starch gelatinization by water absorption, C4 to the stability of formed gel, and C5 to starch retrogradation during the cooling process. When a test was performed, six indices were automatically calculated by the Mixolab Profiler tool, i.e., water absorption index (*WAI*), mixing index (*MI*), gluten+ index (*GI*), viscosity index (*VI*), amylase index (*AI*), and retrogradation index (*RI*). These indices were presented on a six-axis graph (“spider web”) [25].

### 2.5. Characterization of Bread Samples

Physicochemical properties of bread samples, i.e., porosity (*PO*), humidity (*HU*), electrical conductivity (*EC*), and pH (*pH*), were determined in triplicate, and their mean values were reported. *PO* was measured according to STAS 91/1983 [26,27], using a metallic cylinder with the height of 6 cm and a diameter of 3 cm. *HU* was determined using a thermobalance Kern DAB 100-3 (Kern, Frankfurt am Main, Germany). *EC* and *pH* of a suspension consisting of a bread sample (5 g) mixed with distilled water (50 mL) heated at 70 °C were measured using an HI 5521 digital benchtop pH-EC-meter (Hanna Instruments, Woonsocket, RI, USA).

Mold formation was qualitatively assessed three and seven days after bread production to evaluate the shelf life of the bread under normal storage conditions. The bread samples were stored in polyethylene food bags, in the dark, at room temperature and a relative air humidity of 45%.

### 2.6. Statistical Analysis

The values of bread physicochemical properties obtained at different levels of process factors, i.e., the percentages of HC and KGM additives in the mixture of flour and both additives (*c_HC_* = 0.79–2.21% and *c_KGM_* = 0.79–2.21% according to the data summarized in Table 1), were processed using CCD [28,29,30]. Single factor ANOVA was used to evaluate if the effect of dough freezing on bread physicochemical properties was significant (*p* < 0.05) or not (*p* ≥ 0.05). Since only two levels of the freezing factor (frozen vs. non-frozen dough) were compared for each formulation, post-hoc tests were not applicable. Other experimental results were presented descriptively due to the limited number of replicates. Statistical analysis was performed using STATISTICA version 10.0 (StatSoft Inc., Tulsa, OK, USA) and Excel (Microsoft 365).

## 3. Results and Discussion

### 3.1. Characterization of HC and KGM Additives

#### 3.1.1. SEM-EDX Analysis

**Figure 1:**
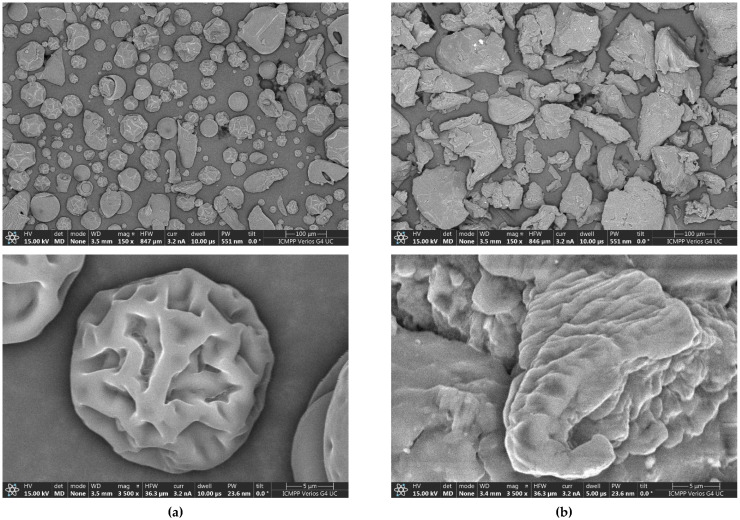
SEM micrographs of HC (**a**) and KGM (**b**) additives.

The SEM images of HC and KGM, which are presented in Figure 1 (100 μm and 5 μm), show the difference between the structural morphologies of these two additives, i.e., HC has the structure of a protein, whereas KGM has the structure of a polysaccharide. In the case of the HC sample, SEM images indicate a porous structure determined by the presence of low molecular weight polypeptides [31]. In contrast, the KGM sample presents particles with a more irregular morphology, having a layered appearance [32]. The surfaces are relatively smoother compared to HC, but are composed of multiple overlapping layers, which suggests a lamellar structure [21]. This structural organization indicates an increased water retention capacity, a specific property of hydrophilic polysaccharides [22]. The differences between the structures of HC and KGM samples are also highlighted by the elemental analysis of both additives, which is presented in Table 2. HC is a protein that contains more N and less C and O than the polysaccharide KGM.

#### 3.1.2. MALDI-TOF MS Analysis

**Figure 2:**
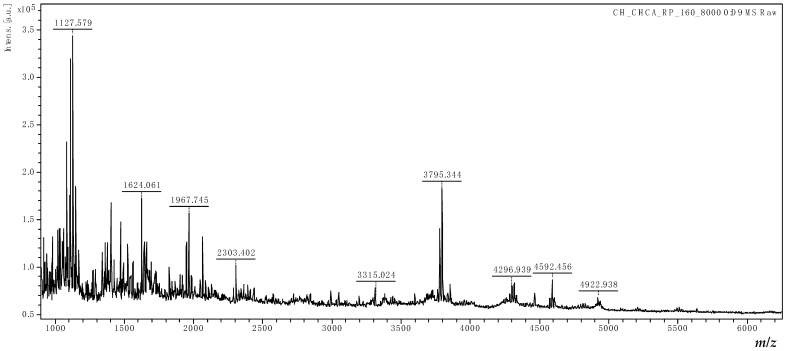
MALDI-TOF mass spectrum of HC.

**Figure 3:**
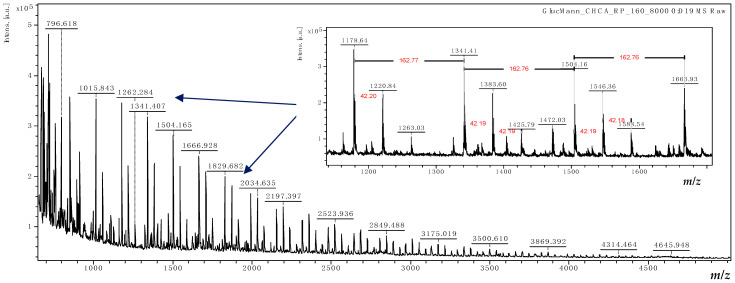
MALDI-TOF mass spectrum of KGM.

The molecular mass (MM) distributions of relevant compounds from HC and KGM were estimated based on MALDI-TOF mass spectrometry. The MALDI-TOF mass spectra of HC and KGM are shown in Figure 2 and Figure 3.

HC contains a group of bioactive di and tripeptides with low MMs (2–6 kDa) that can be obtained by breaking down collagen protein through a hydrolysis process. The specific peptides found in HC can vary depending on the source of the collagen (e.g., bovine, porcine, marine) and the method of hydrolysis [18]. The analyzed peptides are rich in amino acids such as glycine, proline, and hydroxyproline (Gly, Pro, and Hyp), with characteristic sequences like Gly-Pro-X and Gly-X-Hyp (X could be any of the 17 other amino acids). The mass spectrum of used HC (Figure 2) indicates that the MMs of the most abundant peptides are in the range 1–3 kDa, and no signals are obtained above *m*/*z* = 6000. The main peaks correspond to peptide fragments, likely containing multiple repeats of the Gly-Pro-Hyp sequence. For example, the peak at *m*/*z* = 1128 can be attributed to four sequences of Gly-Pro-Hyp (noted as M) as a sodium ion adduct [4M − 2H_2_O + Na]^+^. Also, the peaks at *m*/*z* = 3795 and *m*/*z* = 4923 can be assigned to [14M − 11H_2_O + H]^+^ and [18M − 12H_2_O + Na]^+^ species, corresponding to seven and nine sequences of Gly-Pro-Hyp-Gly-Pro-Hyp, respectively. Based on the results of the MALDI-TOF MS analysis, it can be confirmed that the HC contains very small peptide sequences with an MM of approximately 3 kDa, which proves the high quality of the raw material.

KGM is a polysaccharide consisting of several sugar rings that form linear glycans made up of *D*-mannose and *D*-glucose at a molar ratio of 1:1.6–1:1.4, linked through β-1,4-glycosidic bonds. Additionally, it can contain traces of acetyl groups randomly present at the C6 position and several short mannose branches at the C3 positions [33,34]. The MALDI-TOF mass spectrum of the KGM sample (Figure 3) shows a series of hexose oligomers from 5H to 28H (where H represents hexose units) with mass peaks [nH + H]^+^ from *m*/*z* = 811 to *m*/*z* = 4699. In addition, the presence of one to three acetyl groups attached to the hexose oligomers can be observed. For example, the peaks at *m*/*z* = 1179 can be assigned to [7H + acetyl + H]^+^, while the peaks at *m*/*z* = 1221 and *m*/*z* = 1263 correspond to the seven hexose oligomers with two and three acetyl groups. In the insert in Figure 3, the peak-to-peak mass differences of 163 kDa representing the hexosyl units and 42 kDa specific to the acetyl substitution of oligosaccharides are highlighted.

#### 3.1.3. TGA and DSC Analysis

**Figure 4:**
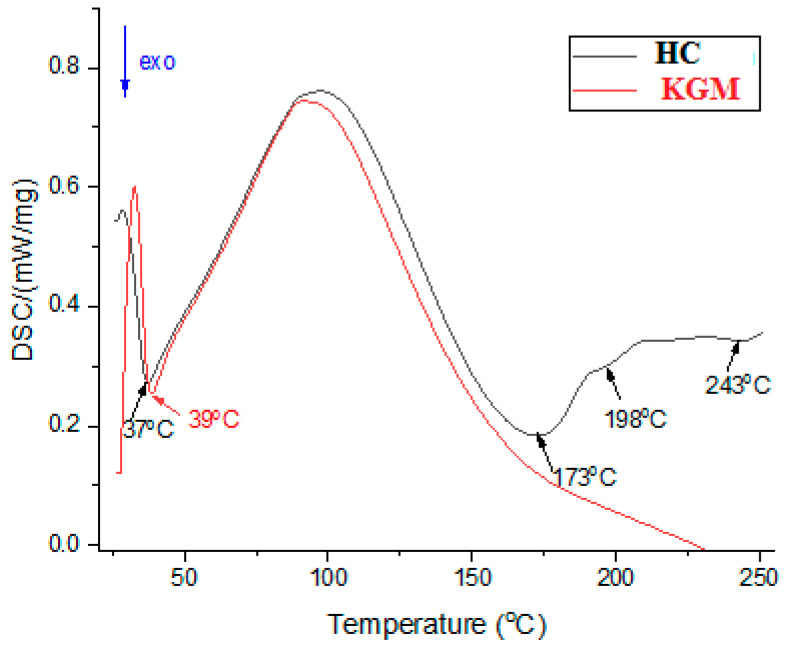
DSC curves of HC and KGM.

The calorimetric analysis confirms the presence of moisture in the HC and KGM samples, with large endothermic peaks being observed around 100 °C in both samples (Figure 4). The presence of water has a good impact on the quality of the bread. Previous results highlighted that the addition of collagen peptides improved the water retention of bread, increased bread specific volume, and slowed down the staling of bread [33,35].

**Figure 5:**
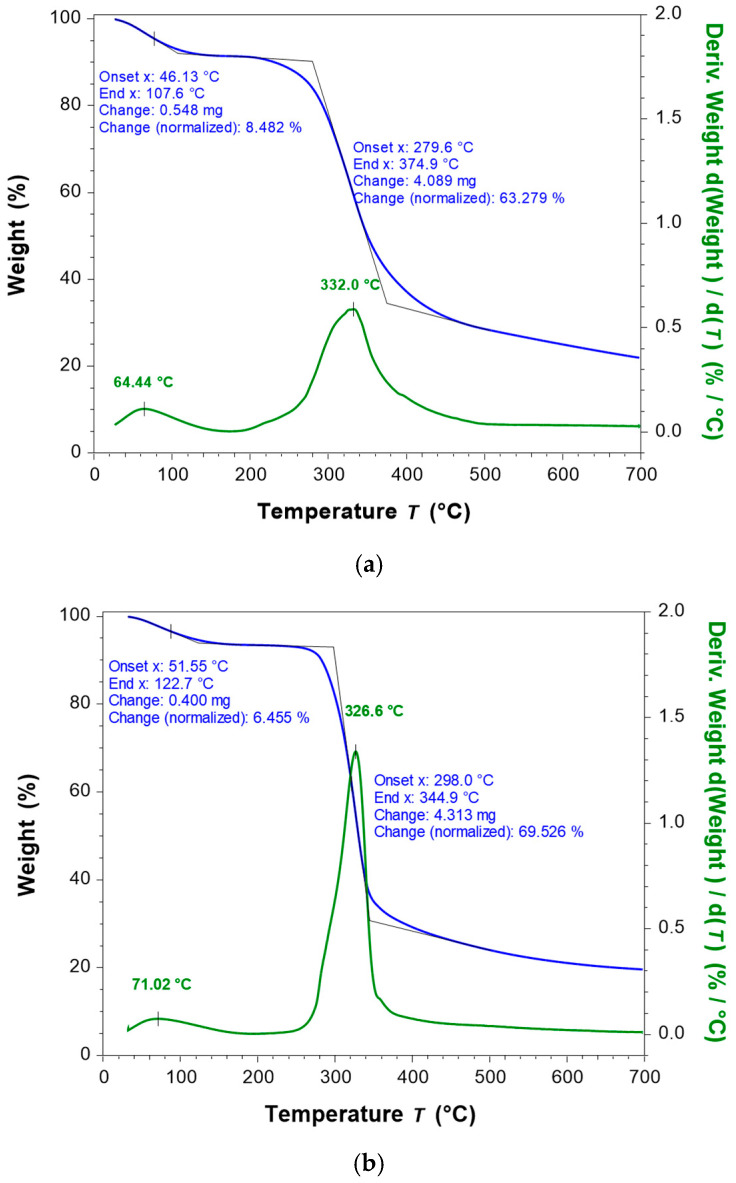
TGA and DTG curves of HC (**a**) and KGM (**b**).

The results of thermogravimetric analysis (TGA) and differential TGA (DTG) are presented in Figure 5. The first peaks observed for both additive samples before 100 °C (*T_peak_*_1_ = 64.4 °C for HC and *T_peak_*_1_ = 71.0 °C for KGM) are mainly attributed to water loss (dehydration). As can be seen in Figure 5, the sample mass loss is 8.48% for HC (*T_onset_*_1_ = 46.1 °C ≤ *T* ≤ *T_end_*_1_ = 107.6 °C) and 6.46% (*T_onset_*_1_ = 51.6 °C ≤ *T* ≤ *T_end_*_1_ = 122.7 °C) for KGM. The second peaks (*T_peak_*_2_ = 332.0 °C for HC and *T_peak_*_2_ = 326.6 °C for KGM), corresponding to mass losses of 63.3% for HC (*T_onset_*_2_ = 279.6 °C ≤ *T* ≤ *T_end_*_2_ = 379.9 °C) and 69.5% (*T_onset_*_2_ = 298.0 °C ≤ *T* ≤ *T_end_*_2_ = 344.9 °C) for KGM, are associated with the decomposition of HC and KGM fibers. At the end of the analysis, a final residue of 20% remained for both samples. TGA and DTG results indicate that both materials exhibited thermal stability up to about 280–296 °C, but differences in *T_onset_*, *T_peak_*, *T_end_*, and mass losses suggest distinct structural features between proteins and polysaccharides. The results obtained in this study are consistent with those reported in the related literature [36,37,38,39,40].

### 3.2. Rheological Properties of Dough Samples

**Figure 6:**
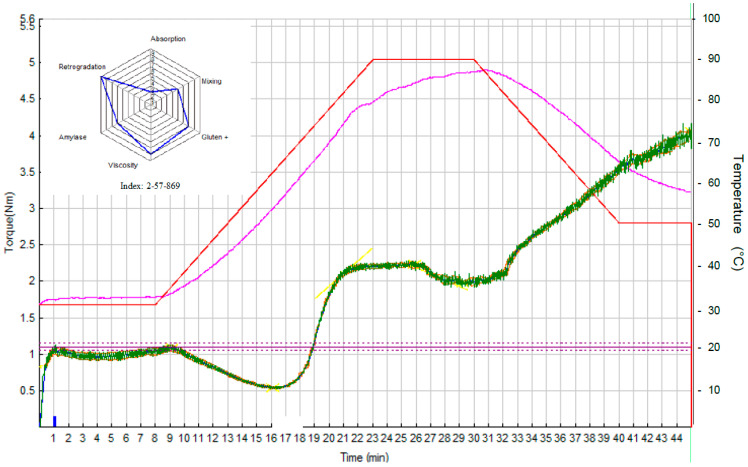
Mixolab curve and Profiler chart for NFD sample S0 (*c_HC_* = *c_KGM_* = 0%).

**Figure 7:**
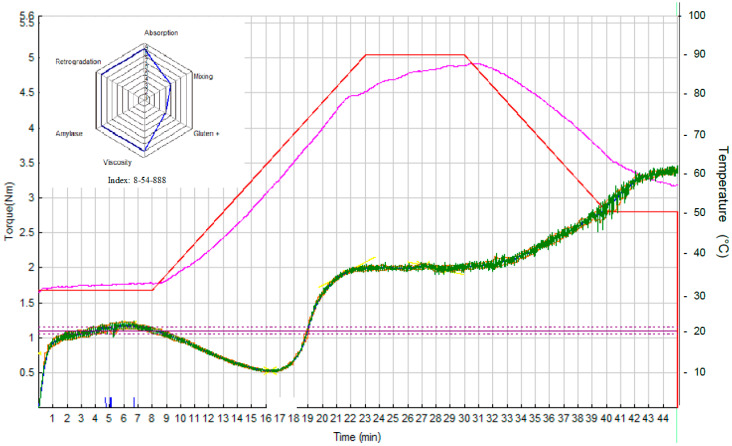
Mixolab curve and Profiler chart for NFD sample S7 (*c_HC_* = 0.79% and *c_KGM_* = 1.50%).

**Figure 8:**
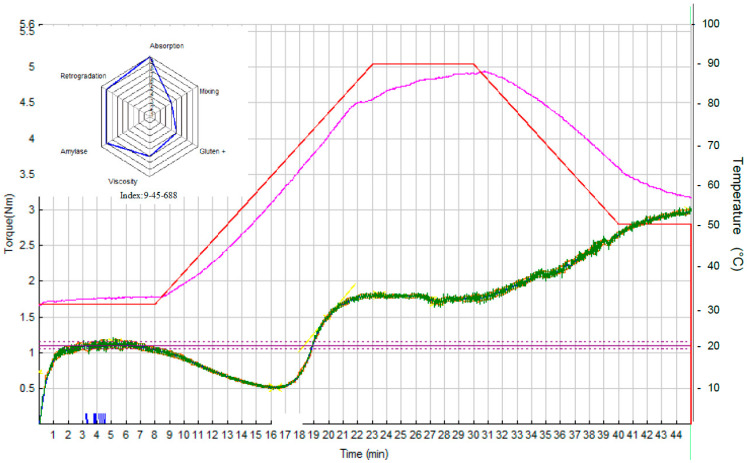
Mixolab curve and Profiler chart for NFD sample S10 (*c_HC_* = 1.50% and *c_KGM_* = 2.21%).

Mixolab curves and Profiler charts (containing six indices, i.e., *WAI*, *MI*, *GI*, *VI*, *AI*, and *RI*) for three samples of NFD, i.e., S0 (*c_HC_* = *c_KGM_* = 0%), S7 (*c_HC_* = 0.79% and *c_KGM_* = 1.50%), and S10 (*c_HC_* = 1.50% and *c_KGM_* = 2.21%), are presented in Figure 6, Figure 7 and Figure 8.

#### 3.2.1. Water Absorption Index (*WAI*)

*WAI* is the simplest and most direct index. The software evaluates the water quantity needed to reach maximum torque C1 at 1.1 Nm (standard method) [25]. An increase in *WAI* (2, 8, and 9 for samples S0, S7, and S10) with an increase in the amounts of HC and KGM (Figure 6, Figure 7 and Figure 8) was observed. On the one hand, the numerous hydroxyl groups of KGM favor water binding through the formation of hydrogen bonds [33,41,42]. Zhou et al. (2014) reported a linear increase in water absorption with an increase in *c_KGM_* from 1% to 5% [42]. On the other hand, peptides in HC can also interact with water, causing an increase in *WAI* [18,35].

#### 3.2.2. Mixing Index (*MI*)

Depending on its rheological strength, dough can exhibit very different behaviors and resistance to mixing stress. The more resistant the dough is to mixing stress, the higher the *MI* will be. The differences between the values of *MI* of the dough samples were not significant (5, 5, and 4 for samples S0, S7, and S10 in Figure 6, Figure 7 and Figure 8), thus the mixing behavior was not influenced by the addition of HC and KGM.

#### 3.2.3. Gluten+ Index (*GI*)

The dough was subjected to two competing stresses: continuous mixing (mechanical stress) and increasing temperature (thermal stress). The decrease in slope is the resulting effect of heat-induced loosening of the gluten proteins. The phenomenon is thermo-reversible and is based only on proteins, since starch gelatinization has not yet started. The results shown in Figure 6, Figure 7 and Figure 8 indicate that the addition of HC and KGM determined a decrease in *GI* (from 7 for sample S0 to 4 and 5 for samples S7 and S10, respectively).

KGM can disrupt the protein matrix of bread dough, leading to a decrease in *GI* and an alteration of the textural properties of the dough [41,42]. The molecules of the two gluten proteins, i.e., glutenin and gliadin, are cross-linked by disulfide bonds (S–S) and non-covalent interactions (hydrogen bonds, ionic bonds, and hydrophobic linkages) to form a continuous three-dimensional network [41]. Zhang et al. (2025) [41] reported that a high concentration of KGM in a flour (*c_KGM_* = 10%) led to the disruption of disulfide bonds between glutenin and gliadin subunits in the strong gluten, causing a decrease in gluten molecular chain height and width (by 74.0% and 38.9%, respectively), a decrease in dough hardness (by 21.6%), and an increase in dough adhesiveness (by 19.1%). The disruption of the cross-linking between strong gluten subunits could be due to the fact that KGM (rich in hydroxyl groups) competed with gluten for the available water, causing a change in the “loop and train” structure of the strong gluten network [41].

#### 3.2.4. Viscosity Index (*VI*)

During this phase, mixing continues and the temperature increases from 60 °C to 80 °C. Several phenomena occur simultaneously in this temperature range, making it difficult to separate individual events. Starch begins to gelatinize around 60 °C, the granules swell, and the dough viscosity increases. It is a major change, as the dough goes from a gluten-supported system to a starch-supported system. Although amylase activity can be observed at lower temperatures, it is generally limited by the semi-crystalline state of the starch. However, amylase reaches a maximum activity at about 60 °C, as starch begins to gelatinize. It is then rapidly inactivated at about 70 °C, and starch hydrolysis stops [25]. So there is a competition between starch gelatinization, which tends to increase dough viscosity, and amylase degradation, which reduces the viscosity. The resulting peak depends on the magnitude of both phenomena. The higher the viscosity, the higher the *VI*. Above a certain amount of HC and KGM, a decrease in *VI* is observed (8 for samples S0 and S7 as well as 6 for sample S10 in Figure 6, Figure 7 and Figure 8). This decrease is probably due to a developed secondary hydrocolloid network that limits the availability of water for starch gelation, which causes a decrease in dough viscosity [18,43].

#### 3.2.5. Amylase Index (*AI*)

*AI* provides a measure of the stability of starch gel in the presence of mechanical stress induced by continuous mixing [25]. If the starch granules have been enzymatically degraded (i.e., long chains broken), the system is more prone to shear thinning. The addition of HC and KGM determined an increase in *AI* (6 for sample S0 and 8 for samples S7 and S10 in Figure 6, Figure 7 and Figure 8). The higher the *AI*, the higher the resistance to amylolysis. Studies in the related literature have indicated that hydrocolloids can reduce starch digestibility by forming networks that limit the attack of amylases on starch granules, which leads to increased stability of the starch gel in the presence of mechanical and enzymatic stresses [44].

#### 3.2.6. Retrogradation Index (*RI*)

The dough was cooled to a temperature of *cca.* 60 °C. As soon as the cooling process begins, an increase in the dough consistency can be observed. When gelatinized starch is cooled, the swollen and/or destroyed starch granules and molecules tend to quickly reform into a crystalline structure [45]. This mechanism is known as starch degradation and is directly related to the shelf life of the product. Of course, degradation is a long process [25]. Recrystallization of amylose occurs rapidly after cooling the gelatinized starch, while recrystallization of amylopectin is a considerably slower process. The results shown in Figure 6, Figure 7 and Figure 8 indicate that the addition of HC and KGM slightly influenced the starch degradation (*RI* = 9 for sample S0 and *RI* = 8 for samples S7 and S10).

### 3.3. Characterization of Bread Obtained from Non-Frozen Dough (NFD) and Frozen Dough (FD)

According to a CCD with two factors and four center point runs, two sets of twelve experimental runs were performed to evaluate the influence of process factors on the relevant characteristics of bread samples prepared from NFD and FD. Percentages of HC and KGM additives in the mixture of flour and both additives (*c_HC_* = 0.79–2.21% and *c_KGM_* = 0.79–2.21%) were chosen as dimensional factors. Dimensionless factors (*x*_1_ and *x*_2_) are defined by Equations (1) and (2). Bread physicochemical properties in terms of porosity (*PO*), humidity (*HU*), electrical conductivity (*EC*), and pH (*pH*) were selected as dependent variables (responses). The values of experimental responses at different levels of dimensional and dimensionless factors, as well as indicators of position (minimum, maximum, and mean values) and variability (standard deviation and coefficient of variation) of selected responses are summarized in Table 3 and Table 4.

(1) $$x_{1}=\frac{c_{HC}-1.5}{0.5}$$

(2) $$x_{2}=\frac{c_{KGM}-1.5}{0.5}$$

The tabulated data highlight a very low variability (0.8% ≤ *CV* ≤ 3.2%) of *PO* for bread prepared from NFD (*PO_NFD_* = 70.18–78.76%) and of *HU*, *EC*, and *pH* for bread samples obtained from both FD and NFD (*HU_FD_* = 43.98–45.60%, *EC_FD_* = 2.08–2.29 μS/cm, *pH_FD_* = 5.5–5.6, *HU_NFD_* = 42.51–44.72%, *EC_NFD_* = 2.06–2.20 μS/cm, and *pH_NFD_* = 5.8–5.9). A data matrix with twelve rows (number of experimental runs) and eight columns (number of responses for bread samples prepared from both FD and NFD, i.e., *PO_FD_*, *HU_FD_*, *EC_FD_*, *pH_FD_*, *PO_NFD_*, *HU_NFD_*, *EC_NFD_*, and *pH_NFD_*, was used in multivariate analysis. Statistical analysis revealed that only *PO_FD_*, *pH_FD_*, and *pH_NFD_* were significantly affected by the process factors.

The effects of *x*_1_, *x*_1_^2^, *x*_2_, *x*_2_^2^, and *x*_1_*x*_2_ on predicted (calculated) responses (*y_j_*, *j* = 1…3) were quantified using second-order polynomial models given by Equations (3)–(5), where *y*_1_ = *PO_FD_*_,_*_calc_*, *y*_2_ = *pH_FD_*_,_*_calc_*, and *y*_3_ = *pH_NFD_*_,_*_calc_* are calculated responses.

(3) $$y_{1}=70.80+1.322x_{1}-6.189x_{1}^{2}-1.377x_{2}-0.698x_{2}^{2}-2.700x_{1}x_{2}$$

(4) $$y_{2}=5.600-0.006x_{1}^{2}+0.018x_{2}-0.031x_{2}^{2}+0.050x_{1}x_{2}$$

(5) $$y_{3}=5.900+0.030x_{1}-0.031x_{1}^{2}-0.030x_{2}-0.031x_{2}^{2}-0.025x_{1}x_{2}$$

Regression coefficients in Equations (3)–(5), which were estimated based on experimental data, are presented in Table 5, where significant coefficients are highlighted in bold. Multiple determination coefficient (*R*^2^), adjusted *R*^2^ (*R*^2^*_adj_*), *F* statistic (*F*), and significance *F* (*p*-value for *F*) are also specified in Table 5. Tabulated results reveal the following aspects:

(i) a significant negative effect of *x*_1_^2^ on *y*_1_;
(ii) a significant negative effect of *x*_2_^2^ and a significant positive effect of *x*_1_*x*_2_ on *y*_2_;
(iii) a significant positive effect of *x*_1_ and significant negative effects of *x*_1_^2^, *x*_2_, *x*_2_^2^, and *x*_1_*x*_2_ on *y*_3_;
(iv) a good agreement between experimental and predicted data (*R*^2^ ≥ 0.833, *R*^2^*_adj_* ≥ 0.695, *F* ≥ 6.001, *p* ≤ 0.025).

**Figure 9:**
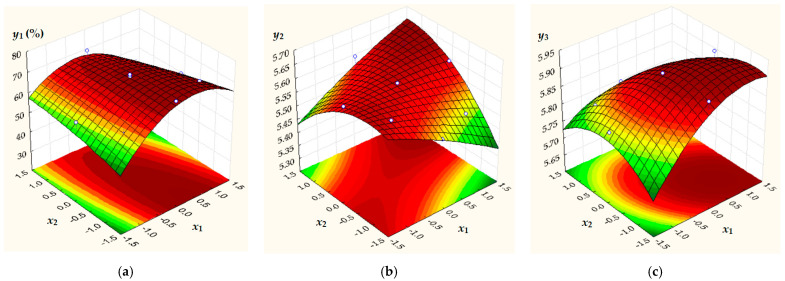
Effects of dimensionless factors *x*_1_ and *x*_2_ on predicted responses: (**a**) *y_1_ = PO_FD_*_,_*_calc_*, (**b**) *y_2_ = pH_FD_*_,_*_calc_*, and (**c**) *y_3_ = pH_NFD_*_,_*_calc_*.

**Figure 10:**
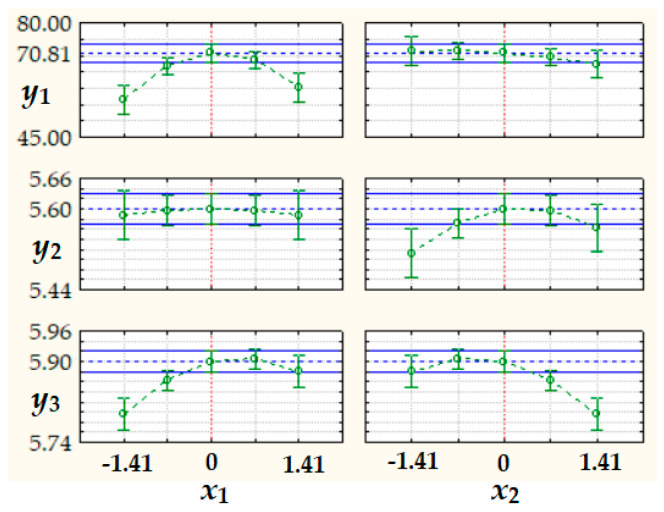
Values of predicted responses (*y_1_ = PO_FD_*_,_*_calc_*, *y_2_ = pH_FD_*_,_*_calc_*, and *y_3_ = pH_NFD_*_,_*_calc_*) at different levels of dimensionless factors *x*_1_ and *x*_2_.

According to the results summarized in Table 5, 3D surface response plots and 2D contour plots of predicted process responses (*y_j_*, *j* = 1…3) depending on dimensionless factors *x*_1_ and *x*_2_ (Figure 9) as well as values of *y_j_* at different levels of *x*_1_ and *x*_2_ (Figure 10) highlight the following:

(i) an increase in *y*_1_ with an increase in *x*_1_ from −1.41 to 0 (*c_HC_* = 0.79–1.50%) followed by a decrease in *y*_1_ with an increase in *x*_1_ from 0 to 1.41 (*c_HC_* = 1.50–2.21%), as well as a non-significant effect of *x*_2_ (*c_KGM_* = 0.79–2.21%) on *y*_1_;
(ii) an increase in *y*_2_ with an increase in *x*_2_ from −1.41 to 0 (*c_KGM_* = 0.79–1.50%) followed by a decrease in *y*_2_ with an increase in *x*_2_ from 0 to 1.41 (*c_KGM_* = 1.50–2.21%), as well as a non-significant effect of *x*_1_ (*c_HC_* = 0.79–2.21%) on *y*_2_;
(iii) an increase in *y*_3_ with an increase in *x*_1_ from −1.41 to 0 (*c_HC_* = 0.79–1.50%) followed by almost constant values of *y*_3_ for *x*_1_ = 0–1.41 (*c_HC_* = 1.50–2.21%); almost constant values of *y*_3_ for an increase in *x*_2_ from −1.41 to 0 (*c_KGM_* = 0.79–1.50%) are followed by a decrease in *y*_3_ with an increase in *x*_2_ from 0 to 1.41 (*c_KGM_* = 1.50–2.21%).

The highest values of predicted responses, i.e., *y*_1_ = *PO_FD_*_,_*_calc_* = 70.81%, *y*_2_ = *pH_FD_*_,_*_calc_* = 5.60, and *y*_3_ = *pH_NFD_*_,_*_calc_* = 5.90, were obtained at *x*_1_ = *x*_2_ = 0 (*c_HC_* = *c_KGM_* = 1.50%). These values of predicted responses were similar with the mean values of experimental responses obtained in the center point runs (*PO_FD_*_,_*_m_* = 70.80%, *pH_FD_*_,_*_m_* = 5.60, and *pH_NFD_*_,_*_m_* = 5.90).

Also, the values of *PO*, *HU*, *EC*, and *pH* specified in Table 3 and Table 4 were processed using a single factor ANOVA to evaluate the effect of dough freezing on bread physicochemical properties. The results of the statistical analysis indicated significant effects (*p* < 0.05) of dough freezing on *PO*, *HU*, and *pH*. Accordingly, the freezing led to a decrease in *PO* and *pH* up to 27% and 7%, respectively, and an increase in *HU* up to 5%. The effect of dough freezing on *EC* was not statistically significant (*p* = 0.10).

**Figure 11:**
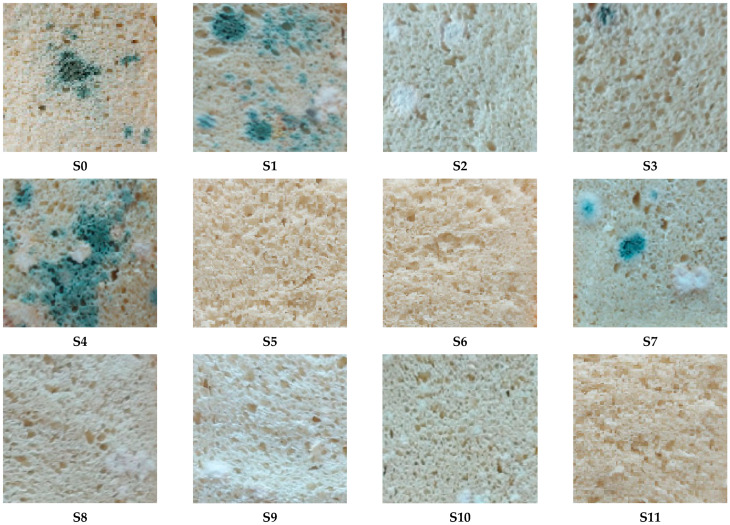
Images of sections of bread samples prepared from NFD samples S0 (*c_HC_* = *c_KGM_* = 0%), S1 (*c_HC_* = *c_KGM_* = 1%), S2 (*c_HC_* = 1% and *c_KGM_* = 2%), S3 (*c_HC_* = 2% and *c_KGM_* = 1%), S4 (*c_HC_* = *c_KGM_* = 2%), S7 (*c_HC_* = 0.79% and *c_KGM_* = 1.50%), S8 (*c_HC_* = 2.21% and *c_KGM_* = 1.50%), S9 (*c_HC_* = 1.50% and *c_KGM_* = 0.79%), S10 (*c_HC_* = 1.50% and *c_KGM_* = 2.21%), S5, S6, and S11 (*c_HC_* = *c_KGM_* = 1.5%), three days after production.

The quality of bread samples obtained from NFD with different doses of HC and KGM was evaluated three days after production, under the same storage conditions (Figure 11). The rate of mold formation depends on the dough composition. Bread samples produced from NFD samples S5, S6, and S11 (*c_HC_* = *c_KGM_* = 1.5%) showed a low rate of mold formation.

**Figure 12:**
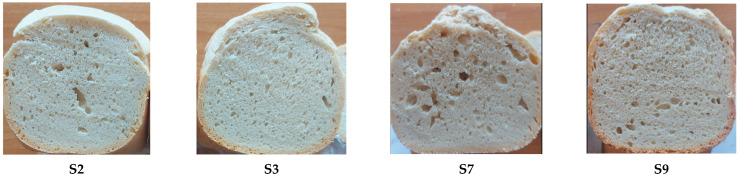
Images of sections of bread samples prepared from FD samples S2 (*c_HC_* = 1% and *c_KGM_* = 2%), S3 (*c_HC_* = 2% and *c_KGM_* = 1%), S7 (*c_HC_* = 0.79% and *c_KGM_* = 1.50%), and S9 (*c_HC_* = 1.50% and *c_KGM_* = 0.79%), three days after production.

Three days after production, all bread samples obtained from FD showed no mold. Images of sections of bread samples prepared from four FD samples, i.e., S2 (*c_HC_* = 1% and *c_KGM_* = 2%), S3 (*c_HC_* = 2% and *c_KGM_* = 1%), S7 (*c_HC_* = 0.79% and *c_KGM_* = 1.50%), and S9 (*c_HC_* = 1.50% and *c_KGM_* = 0.79%), three days after production, are shown in Figure 12. Moreover, bread samples prepared from FD and stored for seven days looked like those obtained from NFD and stored for three days (Figure 11). Accordingly, for bread samples prepared from FD, the mold development process started approximately four days later than for those prepared from NFD. As in the case of bread prepared from NFD, bread samples produced from FD samples in the center point runs (*c_HC_* = *c_KGM_* = 1.5%) showed a low rate of mold formation.

## 4. Conclusions

Frozen doughs (FDs) are increasingly in demand in the bread production process, due to their longer shelf life compared to fresh (non-frozen) doughs (NFDs). However, the big challenge is solving microbiological or rheological problems to improve bread quality by finding the most efficient, sustainable, and ecological additives.

In this study, hydrolyzed collagen (HC) of a bovine origin and bio konjac glucomannan (KGM) were used as biocompatible and low-cost additives in NFD and FD. HC and KGM were characterized using specific analyses, including SEM-EDX, MALDI-TOF MS, TGA, and DSC. The effects of percentages of HC and KGM in the mixture of flour and both additives (*c_HC_* = 0.79–2.21% and *c_KGM_* = 0.79–2.21%) on the relevant characteristics of dough and bread samples were evaluated.

Rheological analysis of three NFD samples, i.e., S0 (*c_HC_* = *c_KGM_* = 0%), S7 (*c_HC_* = 0.79% and *c_KGM_* = 1.50%), and S10 (*c_HC_* = 1.50% and *c_KGM_* = 2.21%), which was performed using a Chopin Mixolab Profiler, highlighted the following aspects: (i) higher values for the water absorption index (*WAI*) and amylase index (*AI*), and lower values for the gluten+ index (*GI*) for samples with HC and KGM than for the control sample S0; (ii) a lower value of the viscosity index (*VI*) for sample 10 (having higher values of HC and KGM concentrations) than for the other samples; and (iii) similar values for the mixing index (*MI*) and retrogradation index (*RI*).

According to a CCD with two factors and four center point runs, two sets of twelve experiments were conducted to evaluate the influence of *c_HC_* and *c_KGM_* on the porosity (*PO*), humidity (*HU*), electrical conductivity (*EC*), and *pH* of bread samples prepared from NFD and FD (*PO* = 58.96–78.76%, *HU* = 42.51–45.60%, *EC* = 2.06–2.29 μS/cm, and *pH* = 5.5–5.9). Statistical analysis revealed that only *PO_FD_*, *pH_FD_*, and *pH_NFD_* were significantly affected by the process factors. Second-order polynomial models were used to predict the process responses depending on its factors. The highest values of predicted responses (*PO_FD_*_,_*_calc_* = 70.81%, *pH_FD_*_,_*_calc_* = 5.60, and *pH_NFD_*_,_*_calc_* = 5.90), which were obtained at *c_HC_* = *c_KGM_* = 1.50% (center point runs), were similar with the mean values of experimental responses. Single factor ANOVA indicated that the freezing led to a significant decrease in *PO* and *pH*, as well as a significant increase in *HU*, whereas its effect on *EC* was not statistically significant.

For bread samples prepared from FD, the mold development process started approximately four days later than for those prepared from NFD. Bread samples produced from FD and NFD samples in the center point runs (*c_HC_* = *c_KGM_* = 1.5%) showed a low rate of mold formation. Future studies on the identification and quantitative analysis of microorganisms are needed to complete data on the safety and quality of bread. Moreover, further analyses of bread dough, e.g., Fourier transform infrared spectroscopy (FTIR), SEM-EDX, TGA, and DSC, should be performed to better elucidate the physicochemical mechanisms responsible for its rheological behavior and changes in bread quality.

## Figures and Tables

**Table 1:** Masses (g) of ingredients used in the bread.

Sample	Ingredients						
	WF	HC Powder	KGM Powder	FY	SO	SA	Water
S0	100.00	0	0	3.59	2.87	1.56	62.50
S1	98.00	1	1	3.59	2.87	1.56	62.50
S2	97.00	1	2	3.59	2.87	1.56	62.50
S3	97.00	2	1	3.59	2.87	1.56	62.50
S4	96.00	2	2	3.59	2.87	1.56	62.50
S5	97.00	1.50	1.50	3.59	2.87	1.56	62.50
S6	97.00	1.50	1.50	3.59	2.87	1.56	62.50
S7	97.71	0.79	1.50	3.59	2.87	1.56	62.50
S8	96.29	2.21	2.21	3.59	2.87	1.56	62.50
S9	97.71	1.50	0.79	3.59	2.87	1.56	62.50
S10	96.29	1.50	2.21	3.59	2.87	1.56	62.50
S11	97.00	1.50	1.50	3.59	2.87	1.56	62.50
S12	97.00	1.50	1.50	3.59	2.87	1.56	62.50

WF: wheat flour (minimum value of wet gluten content of 28%; M.P. BANEASA–MOARA, Buftea, Romania); HC: hydrolyzed collagen (90% proteins; manufactured in Spain, packaged and distributed by Deco Italia, Cluj-Napoca, Cluj, Romania); KGM: konjac glucomannan (89.4% fibers; manufactured in China, and packaged and distributed by Deco Italia, Cluj-Napoca, Cluj, Romania); FY: fresh yeast (purchased from Pakmaya, Pașcani, Iași, Romania); SO: sunflower oil (from Romanian market); SA: salt (from Romanian market).

**Table 2:** Elemental analysis for HC and KGM obtained by SEM-EDX analysis.

Element	HC		KGM	
	Weight (%)	Atomic (%)	Weight (%)	Atomic (%)
C	50.8 ± 3.3	61.0 ± 3.7	60.9 ± 4.3	70.8 ± 4.2
N	21.2 ± 4.2	21.6 ± 2.8	3.1 ± 0.7	3.1 ± 0.7
O	18.7 ± 3.6	16.7 ± 2.1	29.4 ± 4.5	25.6 ± 4.0

**Table 3:** Values of physicochemical properties of bread samples prepared from frozen dough (FD) at different levels of dimensional and dimensionless factors.

Run	*c_HC_* (%)	*c_KGM_* (%)	*x*_1_	*x*_2_	*PO_FD_* (%)	*HU_FD_* (%)	*EC_FD_* (μS/cm)	*pH_FD_*
1	1	1	−1	−1	60.10	44.76	2.160	5.6
2	1	2	−1	1	60.80	44.98	2.290	5.5
3	2	1	1	−1	69.40	43.99	2.190	5.5
4	2	2	1	1	59.30	45.19	2.280	5.6
5	1.5	1.5	0	0	70.93	44.79	2.140	5.6
6	1.5	1.5	0	0	69.92	43.98	2.100	5.6
7	0.79	1.5	−1.41	0	58.96	45.33	2.080	5.6
8	2.21	1.5	1.41	0	60.92	44.39	2.090	5.6
9	1.5	0.79	0	−1.41	71.49	45.25	2.120	5.5
10	1.5	2.21	0	1.41	70.35	45.60	2.210	5.6
11	1.5	1.5	0	0	71.20	44.89	2.150	5.6
12	1.5	1.5	0	0	71.15	44.71	2.120	5.6
Minimum values (*MIN*)					58.96	43.98	2.080	5.5
Maximum values (*MAX*)					71.49	45.60	2.290	5.6
Mean values					66.21	44.82	2.161	5.575
Standard deviations (*SD*)					5.522	0.506	0.070	0.045
Coefficients of variation (*CV*) (%)					8.3	1.1	3.2	0.8

**Table 4:** Values of physicochemical properties of bread samples prepared from non-frozen dough (NFD) at different levels of dimensional and dimensionless factors.

Run	*c_HC_* (%)	*c_KGM_* (%)	*x*_1_	*x*_2_	*PO_NFD_* (%)	*HU_NFD_* (%)	*EC_NFD_* (μS/cm)	*pH_NFD_*
1	1	1	−1	−1	76.62	42.51	2.070	5.8
2	1	2	−1	1	77.43	44.72	2.170	5.8
3	2	1	1	−1	78.35	43.25	2.120	5.9
4	2	2	1	1	73.55	43.96	2.200	5.8
5	1.5	1.5	0	0	75.56	43.15	2.120	5.9
6	1.5	1.5	0	0	75.26	42.57	2.060	5.9
7	0.79	1.5	−1.41	0	70.18	43.71	2.140	5.8
8	2.21	1.5	1.41	0	77.59	43.50	2.070	5.9
9	1.5	0.79	0	−1.41	75.33	43.90	2.090	5.9
10	1.5	2.21	0	1.41	78.76	44.38	2.180	5.8
11	1.5	1.5	0	0	75.85	43.74	2.120	5.9
12	1.5	1.5	0	0	75.73	43.64	2.090	5.9
Minimum values (*MIN*)					70.18	42.51	2.060	5.8
Maximum values (*MAX*)					78.76	44.72	2.200	5.9
Mean values					75.85	43.59	2.119	5.858
Standard deviations (*SD*)					2.316	0.653	0.046	0.051
Coefficients of variation (*CV*) (%)					3.1	1.5	2.2	0.9

**Table 5:** Regression coefficients (*β_kj_*, *k* = 1…6, *j* = 1…3) in Equations (3)–(5), multiple determination coefficient (*R*^2^), adjusted *R*^2^ (*R*^2^*_adj_*), F statistic (*F*), and significance *F* (*p*-value for *F*).

*j*		1	2	3
Response		*y*_1_ = *PO_FD,calc_*	*y*_2_ = *pH_FD,calc_*	*y*_3_ = *pH_NFD,calc_*
*k*	Regressor	*β_k_*_1_	*β_k_*_2_	*β_k_*_3_
1	Intercept	**70.80**	**5.600**	**5.900**
2	*x*_1_	1.322	0	**0.030**
3	*x*_1_^2^	**−6.189**	−0.006	**−0.031**
4	*x*_2_	−1.377	0.018	**−0.030**
5	*x*_2_^2^	−0.698	**−0.031**	**−0.031**
6	*x*_1_ *x*_2_	−2.700	**0.050**	**−0.025**
*R* ^2^		0.910	0.833	0.942
*R* ^2^ *_adj_*		0.835	0.695	0.895
*F*		12.15	6.001	19.67
*p* (significance *F*)		0.004	0.025	0.001

Statistically significant coefficients (*p_kj_* ≤ *α* = 0.05) are written in bold.

## Data Availability

The original contributions presented in this study are included in the article. Further inquiries can be directed to the corresponding authors.
